# Li[Li_0.2_Ni_0.16_Mn_0.56_Co_0.08_]O_2_ Nanoparticle/Carbon Composite Using Polydopamine Binding Agent for Enhanced Electrochemical Performance

**DOI:** 10.1186/s11671-015-0986-0

**Published:** 2015-06-26

**Authors:** Suk Bum Lim, Yong Joon Park

**Affiliations:** Department of Advanced Materials Engineering, Kyonggi University, San 94-6, Yiui-dong, Yeongtong-gu, Suwon, Gyeonggi-do 443-760 Republic of Korea

**Keywords:** Cathode, Lithium battery, Super P, Composite, Polydopamine

## Abstract

Li[Li_0.2_Ni_0.16_Mn_0.56_Co_0.08_]O_2_ nanoparticles were composited with carbon (Super P) in order to achieve an enhanced rate capability. A polydopamine pre-coating layer was introduced to facilitate the adhesion between Super P and pristine nanoparticles. The Super P particles were dispersed on the surface of Li[Li_0.2_Ni_0.16_Mn_0.56_Co_0.08_]O_2_ powders. The composite samples that were heat-treated in a N_2_ atmosphere showed increased capacity and enhanced rate capability, which was caused by the improved electronic conductivity owing to the presence of carbon. However, the composite samples that were heat-treated in air did not present these carbon-related effects clearly. The capacity changes observed during the first several cycles may be due to the oxygen deficiency of the structure caused by the heat-treatment process.

## Background

Nowadays, lithium-ion batteries are being used in emerging applications such as electric vehicles, smart mobile devices, and energy storage systems [1–9]. In order to meet the demands of such applications, better cathode materials capable of delivering more energy for advanced lithium-ion batteries are required [10–15]. Among the cathode materials reported so far, lithium-rich layered oxides, Li[Ni_x_Li_1/3-2x/3_Mn_2/3-x/3_]O_2_, are some of the most attractive candidates because of their high specific capacity and low cost [16–24]. However, these materials suffer from several problems, such as insufficient cyclic performance due to phase instability and low rate capabilities caused by low electronic and ionic conductivities [25, 26]. As an approach to enhance the rate capability, carbon has been coated on the cathode surfaces. In particular, carbon layers prepared by the in situ carbonization of an organic precursor have been successfully applied to olivine iron-based phosphates such as LiFePO_4_ [27–29]. However, a similar method is difficult to apply to lithium-rich layered oxides because in situ carbonization requires heat treatment in an inert atmosphere, which may deteriorate the structural integrity of the lithium-rich layered oxides owing to loss of oxygen during the heating process.

Herein, we prepared a composite material using existing carbon powders in order to improve the rate capability of lithium-rich layered oxides. Li[Li_0.2_Ni_0.16_Mn_0.56_Co_0.08_]O_2_ nanoparticles, which were prepared via a combustion method, were used as lithium-rich layered oxides. Commercially available Super P was adopted as the carbon material. A special point to be considered in this work is that the pristine (uncoated) powders used were characterized by small-sized and differently shaped grains. Moreover, the Super P particles were also extremely small (0.05–0.1 μm), assuring that a homogeneous mixing and composition between cathode powders and Super P is very difficult to achieve. To overcome this problem, a polydopamine pre-coating layer was introduced as a binding agent between the Li[Li_0.2_Ni_0.16_Mn_0.56_Co_0.08_]O_2_ nanoparticles and Super P. The polydopamine coating layer significantly promotes secondary surface-mediated reactions [30, 31], which have been successfully applied to the composition process between carbons and oxides [32–34]. Hence, the polydopamine pre-coating layer on the surface of Li[Li_0.2_Ni_0.16_Mn_0.56_Co_0.08_]O_2_ nanoparticles may facilitate the homogeneous adhesion of Super P particles. The Li[Li_0.2_Ni_0.16_Mn_0.56_Co_0.08_]O_2_/Super P composition is expected to present an enhanced rate capability due to the high electronic conductivity of surface-attached Super P particles.

## Methods

Li[Li_0.2_Ni_0.16_Mn_0.56_Co_0.08_]O_2_ nanoparticles were prepared using a surfactant-modified combustion method. Manganese acetate tetrahydrate [Mn(CH_3_CO_2_)_2_·4H_2_O (Aldrich, 99+%)], nickel(II) nitrate hexahydrate [Ni(NO_3_)_2_·6H_2_O (Aldrich, 99.99 %)], cobalt(II) nitrate hexahydrate [Co(NO_3_)_2_·6H_2_O (Aldrich, 98 %)], lithium acetate dihydrate [CH_3_CO_2_Li·2H_2_O (Aldrich, 98 %)], and lithium nitrate [LiNO_3_ (Aldrich)] were used as source materials. Two types of polymeric materials, gelatin (Aldrich) and hydroxypropylcellulose (HPC, Aldrich), were used as surfactants to control the particle sizes of the cathode materials. The source materials were dissolved in a solvent composed of distilled water and acetic acid. For every 10 g of source material, 1 g of surfactant was added to each solution. The solutions were continuously stirred on a hot plate at 90–110 °C. As the solvent evaporated, the mixed solutions turned into viscous gels. These gels were then annealed at 400 °C for 1 h, during which a vigorous decomposition process occurred, resulting in the formation of an ash-like powder. This powder was ground and then annealed in air, first at 500 °C for 4 h and subsequently at 800 °C for 6 h. Next, the powder was quenched at room temperature.

**Fig. 1:**
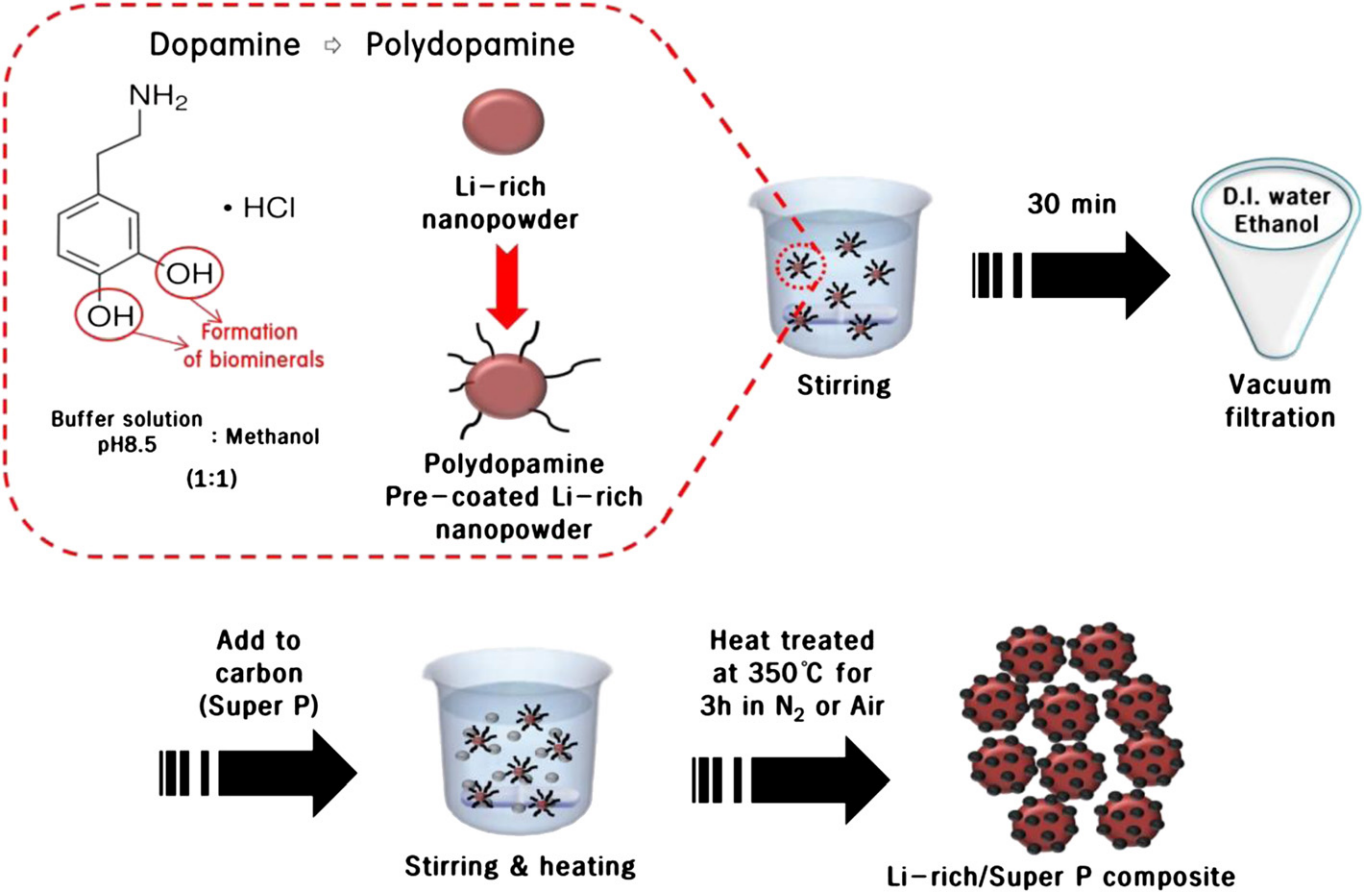
Schematic diagram of the procedure used for the synthesis of the Li[Li_0.2_Ni_0.16_Mn_0.56_Co_0.08_]O_2_/Super P composite using a polydopamine pre-coating layer

To prepare the polydopamine pre-coating layer, each of the two pristine powders was mixed with a dopamine (Aldrich) solution containing Tris buffer (10 mM; pH 8.5; Aldrich) and methanol (Aldrich, 99.9 %) as co-solvents (CH_3_OH:Tris buffer = 1:1 vol%). Through this mixing process, the polymerization process occurred, and a polydopamine layer formed on the surface of pristine powders. The mixture was mechanically stirred at room temperature until all particles were suspended in the solution. Then, the mixture was centrifuged, washed several times with ethanol and distilled water, and dried at 90 °C for 24 h [12, 21, 30–34]. The polydopamine pre-coated powder was added to the carbon (Super P) solution and stirred at 70 °C until all of the solvent evaporated. The carbon content was adjusted to 3 wt% cathode powder. The sample was then dried at 90 °C for 24 h and heat-treated at 350 °C for 3 h in either a N_2_ atmosphere or an air atmosphere. The polydopamine pre-coating layer on the powder surface was removed during the annealing process. Figure 1 shows the scheme used for the synthesis of the Li[Li_0.2_Ni_0.16_Mn_0.56_Co_0.08_]O_2_ nanoparticles/Super P composite using the polydopamine pre-coating layer. The surface morphologies of the samples were analyzed using field emission scanning electron microscopy (FE-SEM, Nova Nano 200). For electrochemical testing, a slurry was prepared by mixing the cathode composite powder with carbon black (Super P) and polyvinylidene fluoride (PVDF) at a weight ratio of 80:10:10 for the cathode composite powder, Super P, and PVDF, respectively. A coin-type cell (2032) configuration composed of a cathode, a Li-metal anode, a separator, and an electrolyte was used. The electrolyte used was a 50:50 vol.% mixture of LiPF_6_ (1 M) and ethylene carbonate/dimethyl carbonate (EC/DMC). Impedance measurements were carried out using an electrochemical workstation (AMETEK, VersaSTAT 3) by applying an AC voltage with an amplitude of 5 mV over a frequency range of 0.1 to 100 kHz.

## Results and Discussion

**Fig. 2:**
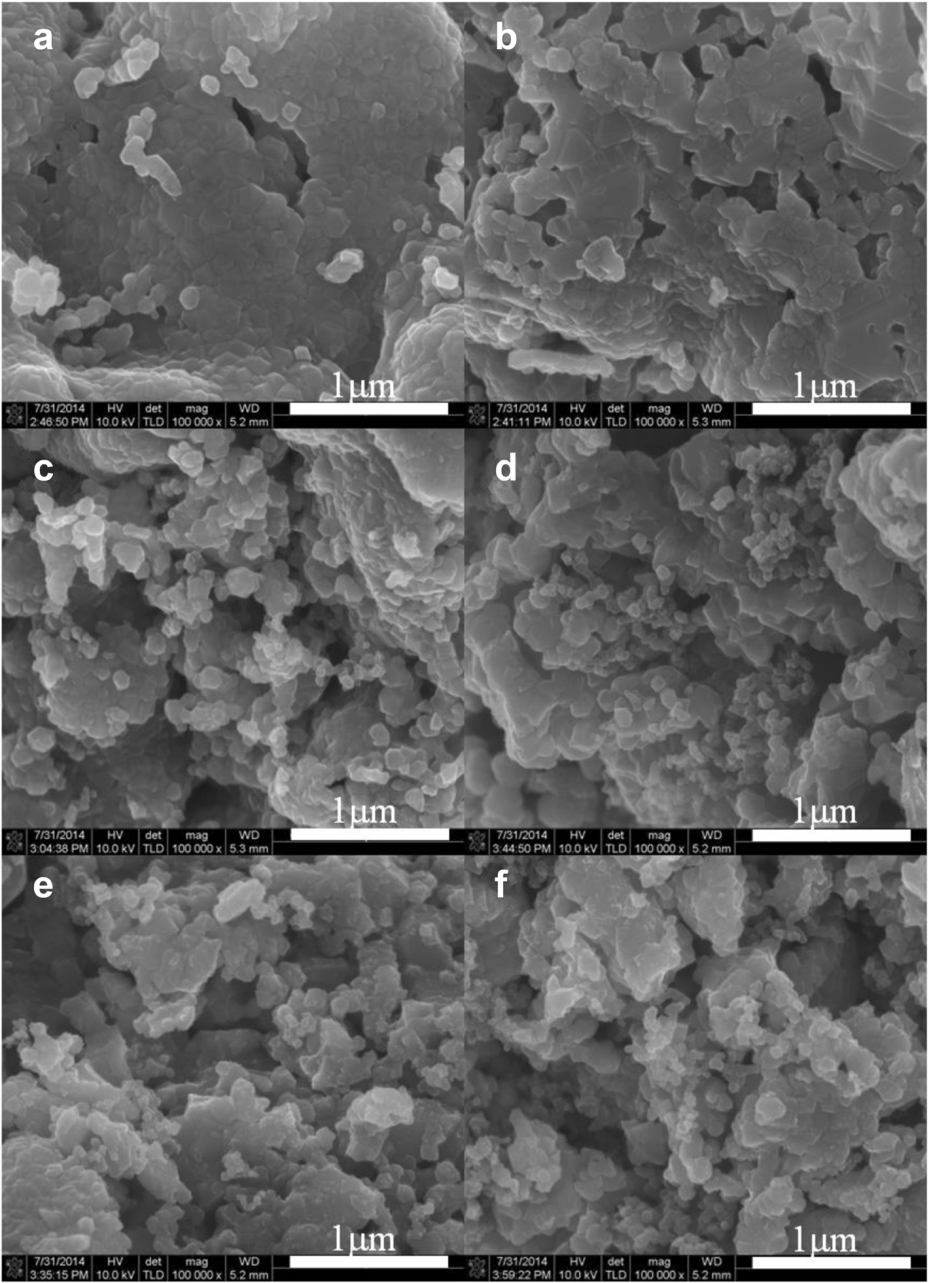
SEM images of the samples: **a** pristine H, **b** pristine G, **c** composite HA, **d** composite GA, **e** composite HN, and **f** composite GN

Figure 2 presents the scanning electron microscopy (SEM) images of the pristine and Super P composite Li[Li_0.2_Ni_0.16_Mn_0.56_Co_0.08_]O_2_ powders. For convenience, hereafter, we refer to the pristine Li[Li_0.2_Ni_0.16_Mn_0.56_Co_0.08_]O_2_ nanoparticles prepared using HPC as ‘pristine H,’ while pristine Li[Li_0.2_Ni_0.16_Mn_0.56_Co_0.08_]O_2_ nanoparticles prepared using gelatin are referred to as ‘pristine G.’ In addition, the different Super P composite particles are referred to as ‘composite HA’ (pristine H/Super P composition heat-treated in air), ‘composite HN’ (pristine H/Super P composition heat-treated in N_2_), ‘composite GA’ (pristine G/Super P composition heat-treated in air), and ‘composite GN’ (pristine G/Super P composition heat-treated in N_2_). As shown in Fig. 2a, b, the pristine H and G powders consist of aggregated primary nanoparticles, while their surfaces are relatively smooth. In contrast, the Super P composite powders exhibit rough surfaces, as shown in Fig. 2c–f. In contrast to the pristine powders, the surface of the composite powder was covered with nano-sized particles, which are expected to correspond to Super P. The nanoparticles seemed to be homogeneously dispersed on the surface of the pristine powder. Furthermore, no special differences were observed between the SEM images of the composite samples.

**Fig. 3:**
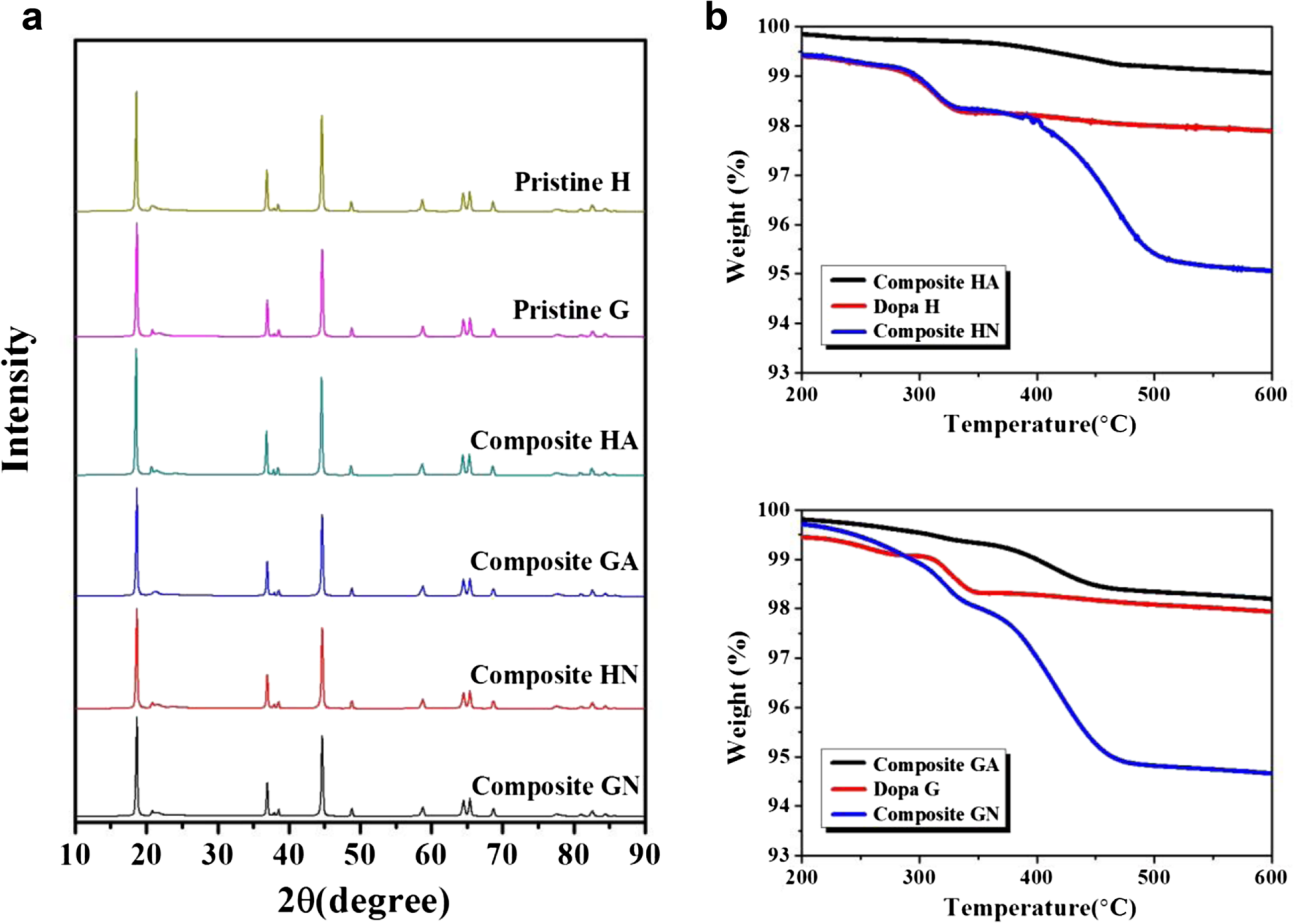
**a** XRD patterns of the pristine and composite samples; **b** TGA results for the samples

Lithium-rich layered oxides have vulnerable phase stabilities; hence, the phase of pristine Li[Li_0.2_Ni_0.16_Mn_0.56_Co_0.08_]O_2_ could change during the heat-treatment process for composite. To determine the phase of the samples, X-ray diffraction (XRD) patterns of the pristine and composite samples were analyzed. As shown in Fig. 3a, the diffraction patterns of all samples seemed to be very similar. Except for the peaks corresponding to superlattice ordering between 20 and 25°, the diffraction peaks of the six samples can be indexed to those of a hexagonal α-NaFeO_2_ structure (space group R-3m). There is no evidence of a special phase change that could be attributed to the heat-treatment process used to prepare the composites. However, the possibility of a small, XRD-undetectable phase change during the heating process cannot be excluded. TGA was also performed on the composite samples to determine of the actual carbon, as shown in Fig. 3b. Considering the weight loss of the composite from 200 to 600 °C, the carbon contents of the samples were approximately 1, 5, 1.5, and 5 wt% for HA, HN, GA, and GN, respectively. As we expected, the composite samples heat-treated at air had smaller amounts of carbon because carbon evaporated and reacted with oxygen. In contrast, the samples heat-treated in N_2_ contained ~5 wt% carbon. Given that the Super P content of the HN and GN composites was 3 wt% (content of Super P), 2 wt% of carbon originated from the carbonization of polydopamine during heat-treatment. The polydopamine pre-coated pristine H and pristine G (which are referred to as Dopa H and Dopa G, respectively, in Fig. 3b) contained ~2 wt% carbon due to carbonization of polydopamine.

**Fig. 4:**
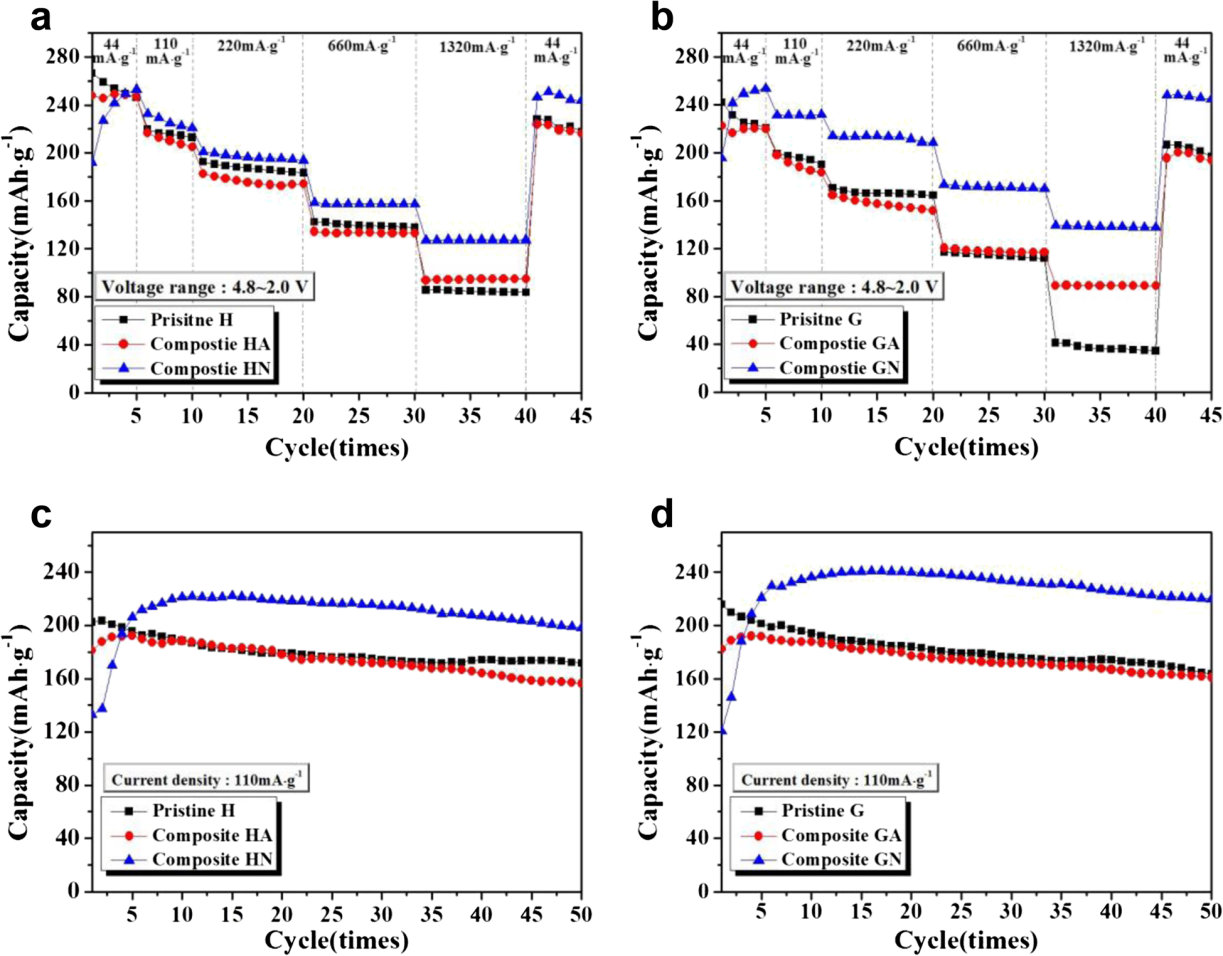
Discharge capacities and cyclic performances of the samples in a voltage range of 4.8–2.0 V; **a** discharge capacity of pristine H, composite HA, and composite HN at current densities of 44, 110, 220, 440, and 1320 mA g^−1^; **b** discharge capacity of pristine G, composite GA, and composite GN at current densities of 44, 110, 220, 440, and 1320 mA g^−1^; **c** cyclic performance of pristine H, composite HA, and composite HN at a current density of 110 mA g^−1^; **d** cyclic performance of pristine G, composite GA, and composite GN at a current density of 110 mA g^−1^

To characterize the effect of the composite with Super P, the electrochemical performance of the pristine and Super P composite samples was evaluated and compared. Figure 4a presents the discharge capacity of pristine H and composites HA and HN measured at current densities of 44, 110, 220, 660, and 1320 mA g^−1^ in a voltage range of 4.8–2.0 V. During the initial five cycles measured at 44 mA g^−1^, the capacity of pristine H seemed to gradually decrease. In contrast, composite HA showed a relatively stable capacity during these initial cycles. The capacity of composite HN started at a much lower value than those of the other samples but rapidly increased during the first five cycles. This change of the initial capacity can likely be ascribed to the effect of the carbon composite and/or thermal treatment during the composition process. After the first five cycles, the capacity of the three samples became very similar. However, at increasing current densities, the composite HN exhibited a significantly enhanced capacity and rate capability when compared to pristine H and composite HA. At a current density of 1320 mA g^−1^, the capacity of composite HN was ~128 mAh g^−1^, while that of the pristine H was only ~85 mAh g^−1^. The capacity retention of composite HN was approximately ~50 % of the capacity measured for a current density of 44 mA g^−1^, which is much higher than that of pristine H (~34 %).

Figure 4b presents the discharge capacity of pristine G, composite GA, and GH measured at various current densities. During the initial five cycles, the discharge capacity of pristine G decreased slightly, similar to that of pristine H. However, composite GA maintained a stable capacity, while composite GN exhibited a rapid increase of capacity, showing similar tendencies as those observed for composite HA and HN, respectively. The rate capabilities of composites GA and GN seemed to be superior to that of pristine G. In particular, composite GN (heat-treated in N_2_) showed a higher capacity and enhanced rate capability compared to the other samples. Table 1 summarizes the discharge capacities and capacity retentions of the samples for all current densities. The values were measured during the first cycle for each current density. Given the results in Fig. 4a, b and Table 1, the composite samples that included Super P and were heat-treated in N_2_ had an enhanced rate capability compared to the pristine samples. When the composite samples were heat-treated in air, the effects caused by the carbon composition seemed to be ambiguous due to the low content of carbon. We adjusted the carbon (Super P) content to 3 wt% of the Li[Li_0.2_Ni_0.16_Mn_0.56_Co_0.08_]O_2_ powder. However, most of the carbon evaporated during the heat-treating process (at 350 °C) in air, as confirmed in Fig. 3b. In contrast, the sample heat-treated in N_2_ had a higher content of carbon, which may have led to the improved rate capabilities for composites HN and GN compared to other samples.

**Table 1:** Discharge capacity and capacity retention of the Li[Li_0.2_Ni_0.16_Mn_0.56_Co_0.08_]O_2_/Super P composite for various current densities. The listed values are obtained from the initial cycle at each current density

	44 mA g^−1^		110 mA g^−1^		220 mA g^−1^		660 mA g^−1^		1320 mA g^−1^	
	(mAh g^−1^)	(%)	(mAh g^−1^)	(%)	(mAh g^−1^)	(%)	(mAh g^−1^)	(%)	(mAh g^−1^)	(%)
Pristine H	246.1	100	212.9	86.5	187.3	76.1	139.3	56.6	84.8	34.4
Composite HA	246.4	100	204.9	83.2	175.2	71.1	133.2	54	94.6	38.4
Composite HN	253	100	221	87.4	196.5	77.7	157.4	62.2	127.5	50.4
Pristine G	220.5	100	190.3	86.3	166.3	75.4	114.8	52.1	36.4	16.5
Composite GA	219.5	100	183.3	83.5	157.1	71.6	117.8	53.6	89.2	40.6
Composite GN	253.6	100	232	91.5	213.7	84.3	171.3	67.6	138.2	54.5

**Fig. 5:**
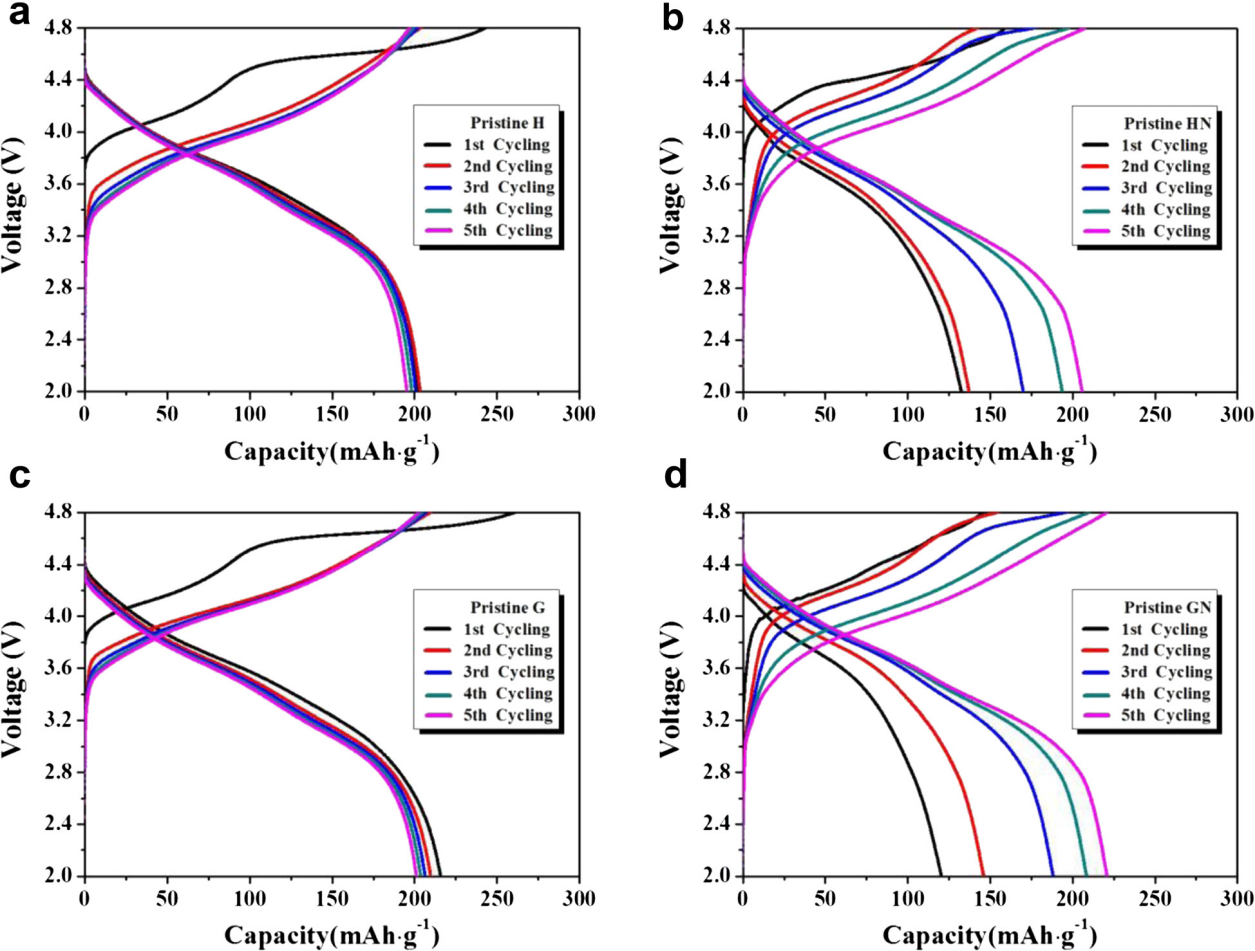
Charge-discharge profiles of the samples during the five initial cycles; **a** pristine H, **b** composite HN, **c** pristine G, and **d** composite GN

Figure 4c, d present the cyclic performance of the samples over a voltage range of 4.8–2.0 V and a current density of 110 mA g^−1^. The capacities of composites HN and GN started at low values. However, these quantities stabilized after several cycles at somewhat higher values than those of pristine H, G and composites HA, GA. This indicates that the composite samples heat-treated in N_2_ had enhanced capacity when compared to other samples. Most carbon in the composite samples that were heat-treated in N_2_ may be still present after the heating process. Hence, the enhanced electronic conductivity of the sample due to the carbon may lead to an increased capacity. In contrast, the samples that were heat-treated in air showed a capacity similar to that of the pristine samples. The rapid increase in capacity found for composites HN and GN is an interesting result, considering that the capacity of the cathode tends to decrease during cycling. Figure 5 compares the charge-discharge profiles of the pristine samples and composites HN and GN during the initial five cycles. As shown in Fig. 5a, c, the first charge profile of the pristine samples shows a long plateau region at around 4.6 V due to the initial irreversible reaction. The irreversible reaction involves the evolution of oxygen in the Li[Li_0.2_Ni_0.16_Mn_0.56_Co_0.08_]O_2_ structure [35]. After the first cycle, the charge-discharge profile of the pristine samples appears to be stable, except for a slight capacity fading during cycling. On the contrary, composites HN and GN did not show a clear plateau region in the first charge profile (Fig. 5b, c). The initial charge-discharge capacity was much smaller than the stabilized capacity. However, those values rapidly increased during cycling. The disappearance of the plateau region may be associated with a decrease in the irreversible reaction that involves the evolution of oxygen during the initial charging process. Composites HN and GN were heat-treated with carbon in an inert atmosphere, so the oxygen in the Li[Li_0.2_Ni_0.16_Mn_0.56_Co_0.08_]O_2_ structure could be released during the heat-treatment process. Thus, composites HN and GN had an oxygen-deficient Li[Li_0.2_Ni_0.16_Mn_0.56_Co_0.08_]O_2_ structure, which may have led to a decrease in the irreversible reaction in the initial charging process. Through the first several cycles, the structure of composites HN and GN seemed to be stable, and their high capacities were maintained up to 50 cycles.

**Fig. 6:**
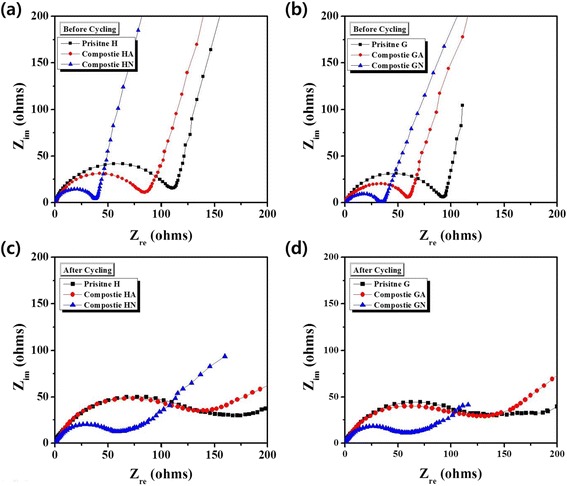
Nyquist plots of the samples: **a** pristine H, and composites HA and HN before electrochemical testing; **b** pristine G, and composites GA and GN before electrochemical testing; **c** pristine H, and composites HA and HN after 50 cycles; **d** pristine G, and composites GA and GN after 50 cycles

An impedance analysis was carried out in order to obtain more information on electrodes containing pristine and composite samples. As shown in Fig. 6a, b, it becomes clear that the impedance spectra of the composite samples showed somewhat smaller semicircles before the test compared with those corresponding to the pristine samples. This implies that the impedance values decreased because of the carbon composition. In particular, composite samples that were heat-treated in N_2_ had a lower impedance value than other samples. After 50 cycles, the impedance values of the samples increased, as shown in Fig. 6c, d. However, composites HN and GN still exhibited lower impedance values compared with other samples such as pristine samples and composites HA and GA. The presented results show that composition with carbon effectively decreased the impedance of the electrode, especially when the carbon composite was heat-treated in N_2_. It can be also inferred that the low impedance of composites HN and GN led to an enhanced rate capability, as shown in Fig. 4a, b.

## Conclusion

In summary, a composite consisting of Li[Li_0.2_Ni_0.16_Mn_0.56_Co_0.08_]O_2_ nanoparticles and carbon (Super P) was prepared in order to improve the rate capability of pristine cathodes. A polydopamine layer was pre-coated on the surface of Li[Li_0.2_Ni_0.16_Mn_0.56_Co_0.08_]O_2_ nanoparticles as a binding agent to promote a homogenous composition with Super P. The Super P nanoparticles were successfully dispersed and attached on the surface of pristine powders. The electrochemical properties of the composites were highly dependent on the heat-treatment atmosphere. The rate capability and discharge capacity were significantly enhanced when the composites were heat-treated in N_2_, which is attributed to the high conductivity of the composite owing to the presence of carbon. However, the composites that were heat-treated in air did not clearly show an improved electrochemical performance. The impedance values of the composite samples (especially those heat-treated in N_2_) were smaller than those of pristine samples both before and after cycling. This result can explain the enhanced rate capability of the composite samples.
